# Nutrient Dosing Framework for an Emission-Free Urban Hydroponic Production

**DOI:** 10.3389/fpls.2021.768717

**Published:** 2021-11-23

**Authors:** Tae In Ahn, Jai-Eok Park, Je Hyeong Jung, Sang Min Kim, Gyhye Yoo, Hyoung Seok Kim, Ju Young Lee

**Affiliations:** Smart Farm Research Center, KIST Gangneung Institute of Natural Products, Gangneung, South Korea

**Keywords:** sustainability, vertical farming, fertilizer, root-zone, hydroponics, urban agriculture

## Abstract

The urban hydroponic production system is accelerating industrialization in step with the potentials for reducing environmental impact. In contrast, establishing sustainable fertilizer dosing techniques still lags behind the pace of expansion of the system. The reproducibility of root-zone nutrient dynamics in the system is poorly understood, and managing nutrients has so far primarily relied on periodic discharge or dumping of highly concentrated nutrient solutions. Here, we assayed root-zone nutrient concentration changes using three possible nutrient dosing types. Three *Brassica* species were hydroponically cultivated in a controlled environment to apply the nutrient absorption and transpiration parameters to the simulation analysis. We found that nutrient dosing based on total ion concentration could provide more reproducible root-zone nutrient dynamics. Our findings highlight the nutrient absorption parameter domain in management practice. This simplifies conventional nutrient management into an optimization problem. Collectively, our framework can be extended to fertilizer-emission-free urban hydroponic production.

## Introduction

Soil-based agriculture is facing substantial challenges such as the loss of arable land, water scarcity, nutrient leaching, and the carbon costs of transporting products. Under these circumstances, as a complementary solution, urban agriculture and vertical farming are currently being explored (Benke and Tomkins, 2017; O’Sullivan et al., 2019). The vertical farming system uses resources independently of the soil surface and expects optimal land use and environmental friendliness. In line with this, the industry is also gearing up. Recently, there has been significant investment in the urban and vertical farming industry. Traditional agribusiness, biotech, and holding companies are entering a global race for new agricultural technologies (O’Sullivan et al., 2019). Inspired by the potential of sustainability, researchers have started to explore urban vertical farming production (Seto and Ramankutty, 2016; Eshed and Lippman, 2019; Eldridge et al., 2020; Kwon et al., 2020; SharathKumar et al., 2020). However, at the same time, there are criticisms about technical pitfalls that prevent vertical farming from substantially contributing to environmental sustainability (Russo and Cirella, 2019). In general, a vertical farming system requires a highly controlled environment in essential factors for plant growth such as light, temperature, water, and mineral nutrients (Kozai and Niu, 2016). The technological status of urban agriculture and vertical farming as a complementary approach to traditional farming is still in its infancy, and there is no solid technological framework for sustainable resource management.

A hydroponic cultivation system, a core technology of urban agriculture, also entails unsolved issues. Inevitably, in hydroponics, fertilizer and water are used intensively. For example, as standard conditions, 2–14 mM of nitrate is prepared in the smaller rooted volume (van Delden et al., 2020). However, still, periodic discharge or dumping of waste fertilizer solution is a trade-off for nutrient management. The repeated emission of concentrated nutrient solutions to the environment has long been an unsolved issue inherent to the conventional greenhouse-based hydroponic sites. Until now, most farms in the countries where there is no legal mandate do not want to take the management risk from blocking the wastewater. In South Korea, hydroponic fertilizers equivalent to nearly 7,400 kg/ha/year are consumed, and 25–30% of the supply is discharged outside the farms (Lee and Kim, 2019).

On the contrary, only about 5% of the farms use wastewater recycling systems. In Almería, Spain, one of the highest areas of greenhouse-based hydroponics, 12% in 3,000 ha of farms uses a recycling system (Massa et al., 2020). This trade-off between the nutrient solution discharge and the nutrient management is related to the concerns about unwanted deviations from standard nutrient conditions and microbial risk. In microbial risk management, a sterilizer could provide a solution (Ahn et al., 2021). On the other hand, in the root-zone, multiple essential nutrients show dynamic changes with perturbations in water absorption, one of the major driving factors of root-zone nutrient variations (Van Noordwijk, 1990; Le Bot et al., 1998). As a result, in hydroponic systems, which are most advantageous for manipulating root-zone nutrition for experiments or cropping, various root-zone nutrient fluctuations have been reported (Zekki et al., 1996; Massa et al., 2011; Signore et al., 2016; Miller et al., 2020).

Due to these seemingly complicated aspects, most hydroponic systems referenced empirical management strategies such as leaching fraction and a suitable period of reuse (Jones, 2005). In rooting medium culture, due to temporal and spatial variations in mass flow, heterogeneity of nutrient distribution remains high (De Rijck and Schrevens, 1998b; Bougoul and Boulard, 2006). Thus, this adds one more layer of complexity to the problem. Instead, in water-based hydroponic cultivation systems, the volume of water and the concentration of nutrients are the most basic and measurable physicochemical factors in root-zone management (Jones, 2005). Thus, root-zone conditioning is inevitably approached based on volume and concentration. In many studies, water level, electrical conductivity (EC, i.e., total ionic concentration), and pH have been used as standard indicators (Fanasca et al., 2006; Signore et al., 2016; Lu et al., 2017). However, it is poorly understood how the nutrient dosing methods could manage the nutrient dynamics. Previous studies have focused on on-line measurements, and several applicable technologies were provided, but it is still challenging to replace conventional nutrient management in terms of technical stability or essential ion measurements (Bratov et al., 2010; Bamsey et al., 2012). These lack of framework for understanding nutrient reproducibility according to nutrient management limits our ability to deduce resource management techniques for zero emissions.

Here we designed a hydroponic simulation model for the Michaelis–Menten equation-based simple hydroponic system with stochastic variations in transpiration rate to address this problem. Three Brassica species (curly kale, lacinato kale, and pakchoi) were hydroponically cultivated in a controlled environment to apply the nutrient absorption and transpiration parameters to the simulation model. Three commonly applicable nutrient dosing methods in the combination of volume, EC, and time were applied in the simulation study. By applying stochastic transpiration variations and the estimated parameters to the hydroponic simulation model with three nutrient dosing methods (volume-, time-, and EC + volume-based dosing), we were able to theoretically predict that the reproducible root-zone nutrient dynamics could be acquired by the EC + volume-basis nutrient dosing method. The reproducible root-zone nutrient dynamics under the stochastic transpiration variations suggest that the root-zone nutrient variations could be managed at the nutrient absorption parameter domain. Our systematic assessment shows that optimization analyses present a novel method to determine the nutrient dosing composition for standard nutrient conditions in the root-zone without periodic dumping or discharge of waste fertilizer solutions.

## Materials and Methods

### Nutrient Absorption Measurement Under a Controlled Environment

Three *Brassica* plant species were used for the nutrient absorption experiment. Pakchoi (*Brassica rapa* subsp. chinensis), lacinato kale (*Brassica oleracea* L., var. acephala), and curly kale (*Brassica oleracea* L., var. sabellica) were cultivated under a controlled environment system at the Korea Institute of Science and Technology (SMART U-FARM, KIST, Gangneung, South Korea). The three *Brassica* species were simultaneously sown and grown hydroponically for 10 days. At 11 days after sowing, 84 plants of each *Brassica* species were selected and transplanted to the hydroponic growing system consisted of a single nutrient container (0.3 m^3^) and cultivated for 54 days. The temperature in the growing room was controlled at 20 ± 1°C (night) and 23 ± 1°C (daytime). LED lamps (KLB-40-2C, red:blue: white = 10:3:2 ratio, KAST Engineering, South Korea) were used for daytime lighting. The LED lamps’ wavelength was composed of red (660 nm), blue (440 nm), and warm white. The average photosynthetic photon flux density (PPFD) in the growing bed was 120 ± 5 μmol m^–2^ s^–1^. Diurnal light conditions were applied (14 h daytime and 10 h dark). The average relative humidity was 73 ± 5% at night and 60 ± 4% at daytime. Carbon dioxide (CO_2_) was supplied at 714 ± 40 ppm.

**FIGURE 1:**
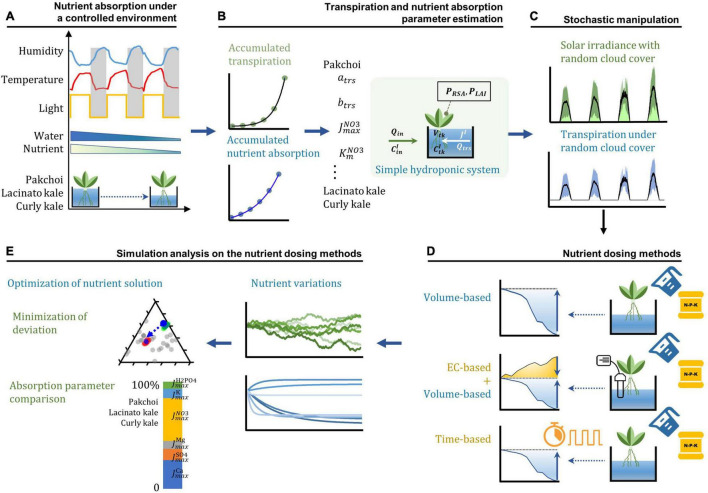
The workflow of the theoretical and experimental analysis setup. **(A)** Nutrient absorption measurement of three *Brassica* species under a controlled environment. **(B)** Parameter estimation process under the simple hydroponic system model by using the progress curve analysis. **(C)** Stochastic manipulation of solar irradiance with a random walk generated cloud cover and subsequent transpiration rate variations in the simple hydroponic system model. **(D)** Three nutrient dosing processes in the simulation analysis. **(E)** Simulation analysis of the nutrient dosing effects on the nutrient variations and nutrient solution optimization.

Macronutrient absorption was calculated by referring to Dr. Yamazaki’s method (N/W) (Wada, 2019). The N/W method calculates the absorption amount based on the container’s initial nutrient concentration and volume and a concentration and a volume of each nutrient at the end of the given period (Figure 1A). On a weekly basis, the initial macronutrients (K, Ca, Mg, NO_3_, H_2_PO_4_, and SO_4_) concentrations were analyzed, and the water volume of the nutrient solution container was measured as the initial condition of the system. For NO_3_^–^, H_2_PO_4_^–^, and SO_4_^2–^ analysis, ion chromatography was performed (730 Professional IC, Metrohm, Switzerland). K^+^, Ca^2+^, and Mg^2+^ were analyzed using an inductively coupled plasma-optical emission spectrophotometer (ICP-OES, PerkinElmer SCIEX, United States). All the analytical procedures were validated using certified internal reference materials and the average concentration values were obtained by three repeated measurements. The replenishment of the nutrient solution during the week was not performed. At the end of the week, the final macronutrient concentrations were analyzed, and the water volume of the nutrient solution container was measured as the final condition of the system. After the final value was measured, the container’s nutrient solution was replaced entirely with the fresh nutrient solution. The initial nutrient condition composition of macro-elements (K, Ca, Mg, NO_3_, H_2_PO_4_, and SO_4_) was established by referring to the percentage equivalent ratios of anions and cations in Steiner’s universal nutrient solution (Steiner, 1980; Jones, 2005). The total ion equivalent concentration of the initial nutrient solution was provided at 26.1 ± 3.8 meq L^–1^ (7.5 ± 1.0, 3.0 ± 1.4, 2.5 ± 1.1, 4.0 ± 0.4, 1.9 ± 0.4, and 1.4 ± 0.2 mM of NO_3_^–^, H_2_PO_4_^–^, SO_4_^2–^, K^+^, Ca^2+^, and Mg^2+^) and the initial nutrient conditions distributing around Steiner’s standard ratio (NO_3_^–^: 60%, H_2_PO_4_^–^: 5%, SO_4_^2–^: 35%, K^+^: 35%, Ca^2+^: 45%, Mg^2+^: 20%) were established to estimate the average level of nutrient absorption parameters. The micronutrients in the nutrient solution were Fe, B, Mn, Cu, Zn, and Mo at 2.80, 0.32, 0.77, 0.04, 0.02, and 0.02 ppm, respectively.

### Hydroponic System Model

In the present study, a simple hydroponic system model was designed. The simulation scale of nutrients and water in the model was taken equal to the experiment’s hydroponic system specification. In the model, we considered nutrient dosing methods, nutrient absorption kinetics, the transpiration rate, and nutrient and water absorption capacity variations due to plant growth.

The nutrient dynamics in the hydroponic system can be expressed as follows:


(1) $$V_{tk}\frac{dC_{tk}^{I}}{dt}=Q_{in}C_{in}^{I}-J^{I}$$

where *V*_*tk*_ is the water volume (m^3^) in the nutrient solution tank of the hydroponic system, *C* is the nutrient concentration (mol m^–3^), superscript *I* is the type of nutrients (K, Ca, Mg, NO_3_, H_2_PO_4_, and SO_4_), and *Q* is the flow rate (m^3^ h^–1^) of water to the nutrient solution tank. Subscript *tk* and *in* represent nutrient solution tank and dosing, respectively, and indicate the variables’ location.

Water volume in the nutrient solution tank of the hydroponic system can be expressed as follows:


(2) $$\frac{dV_{tk}}{dt}=Q_{in}-Q_{trs}$$

where *Q*_*trs*_ is the transpiration rate per plant (m^3^ h^–1^ plant^–1^).

The Michaelis-Menten equation has been used widely in representing the plant’s nutrient absorption behavior (Le Bot et al., 1998). The following equation can express nutrient absorption variation along with plant growth:


(3) $$J^{I}=P_{RSA}\frac{J_{max}^{I}C^{I}}{K_{m}^{I}+C^{I}}$$

where *P*_*RSA*_ is root surface area (m^2^), *J^I^* is the nutrient absorption rate (mol m^–2^h^–1^), $J_{\max}^{I}$ (mol m^–2^ h^–1^) is the maximum absorption rate of nutrient *I*, and $K_{m}^{I}$ (mol) is the Michaelis-Menten parameter. In this model, the nutrient absorption dynamics are described by the function of nutrient concentrations and root growth (Silberbush et al., 2005).

The root surface area *P*_*RSA*_ was assumed to smooth cylinders and calculated with root length and constant mean radius, and the root length variation is modeled using a logistic function of time (Barber, 1995):


(4) $$P_{RL}=\frac{R_{max}}{1+K_{1}e^{-k_{1}t}}$$

(5) $$P_{RSA}=2\pi r_{0}P_{RL}$$

where *P*_*RL*_ is the root length (m), *R*_*max*_ is the maximal root length (m), *K*_1_ and *k*_1_ are coefficients, *r*_0_ is the mean root radius (m), and *t* is the elapsed time (h).

For the transpiration model, the Penman–Monteith equation can be used to predict transpiration by crops.

We used the empirical version of the Penmen–Monteith equation (Bailey et al., 1993; Choi and Shin, 2020). In this model, the transpiration rate is mainly determined by the solar irradiance, the vapor pressure deficit (VPD), and plant growth by the following equation:


(6) $$Q_{trs}=a_{trs}\left(1-e^{-k_{ext}P_{LAI}P_{VPD}}\right)K^{+}+b_{trs}P_{LAI}P_{VPD}$$

(7) $$P_{LAI}=\frac{a_{LAI}}{\left[1+e^{\frac{x_{0}-t}{b_{LAI}}}\right]}$$

where *a*_*trs*_ and *b*_*trs*_ are empirical coefficients, *k*_*ext*_ is the extinction coefficient in the plant canopy, *K*^+^ is the solar irradiance (W m^–2^), *P*_*LAI*_ is the leaf area index (LAI), and *P*_*VPD*_ is VPD (kPa). The Boltzmann sigmoid equation was used to express LAI variations. *a*_*LAI*_, *b*_*LAI*_, and *x*_0_ are the empirical coefficient.

The parameters used in this model were estimated by a progress curve analysis that estimates the value that minimized the root mean square error (RMSE) between the measured and simulated values. In the parameter estimation process, PPFD and relative humidity variations during the day/night cycle were converted to a radiometric unit (W m^–2^) and VPD (kPa). W m^–2^ was obtained by dividing PPFD by the conversion constant of warm-white fluorescent (Thimijan and Heins, 1983). VPD was calculated by the vapor pressure equation (Choi and Shin, 2020). Transpiration and nutrient absorption data measured in the controlled environment condition were applied for the estimation of the parameters (Figure 1B).

### Stochastic Manipulation of Transpiration Rate

Under the hydroponic system model’s verified condition, the stochastic changes in the transpiration rate were applied (Figure 1C). The transpiration model used in this study could be mainly driven by solar irradiance and VPD. Thus, the stochastic seasonal variations in solar radiation and VPD could generate dynamic changes in the transpiration, nutrient concentration, and water content in the hydroponic system. The dynamic changes in incoming solar radiation could be modeled by the total cloud cover model based on solar elevation (Holtslag and Van Ulden, 1983). The framework for stochastic changes in the incoming solar radiation model and VPD was referred to water dynamics model of an automated soilless irrigation system (Ahn et al., 2021). In this model, solar irradiance is the incoming solar radiation at ground level under the total cloud cover, and the dynamic weather changes were simulated by moving the cloud cover in a random walk process. In the case of stochastic changes in VPD, the model simulates a random walk process between 0.5 and 2.0 kPa. However, in this study, the model parameter estimation was conducted under controlled environmental conditions, and the solar irradiance level could largely deviate from the experimental condition. Thus, the solar irradiance was reduced to distribute the simulated transpiration around the measured transpiration rate.

### Simulation Analysis

We examined the reproducibility of the root-zone nutrient dynamics according to the nutrient dosing methods with these models. Three conventional nutrient dosing methods were considered in this analysis: (1) volume-based, (2) time-based, and (3) EC + volume-based (Figure 1D). The volume-based dosing method routinely supplies the standard nutrient solution based on the nutrient solution’s initial volume. The consumed water and nutrients are compensated at the dosing time by supplying the standard nutrient solution to the container’s initial nutrient solution volume. The time-based dosing method supplies a fixed amount of nutrients at a fixed rate. EC + volume-based dosing method estimates total ionic concentration and compensates consumed total mineral nutrients to the initial value, and supplies water to the container’s initial nutrient solution volume.

Under the reproducible nutrient dynamics condition in this simulation analysis, the optimization analysis was conducted to seek the standard nutrient solution for the least deviating from the initial root-zone nutrient condition (Figure 1E). The optimization analysis was carried out in the same way as the parameter estimation by the progress curve analysis in this study. During the process, the molar nutrient concentrations were converted to the percentage molar ratios between the nutrients. Therefore, in this way, it was made to be approached by the scaling relationship of a plant stoichiometric perspective. The mean values of estimated nutrient absorption parameters from three *Brassica* species were converted to the percentage molar ratios and compared in this perspective.

## Results

**FIGURE 2:**
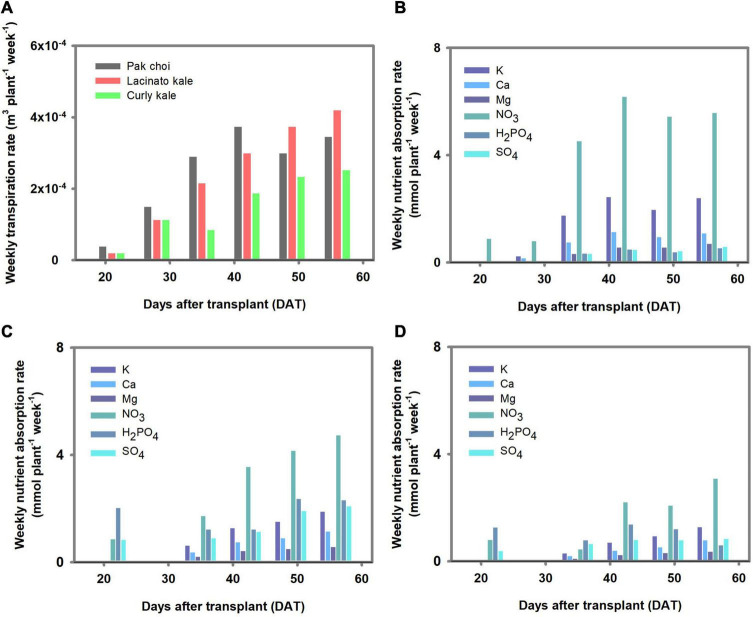
Transpiration and nutrient absorption rate estimated in the controlled environment experiment. **(A)** Weekly transpiration rate of three *Brassica* species. The weekly nutrient absorption rate of pakchoi **(B)**, lacinato kale **(C)**, and curly kale **(D)**.

The overall increase in weekly transpiration and nutrient absorption rate was observed in the three *Brassica* species as the cultivation proceeded (Figure 2). Differences among the three *Brassica* species’ transpiration and nutrient absorption rates were observed to be in the order of pakchoi > lacinato kale > curly kale. NO_3_ was the most absorbed nutrient in all plants. The difference in absorption rate among individual nutrients was similar in all plant species, except SO_4_. SO_4_ absorption amounts were distinctively higher in lacinato and curly kale than in pakchoi.

**FIGURE 3:**
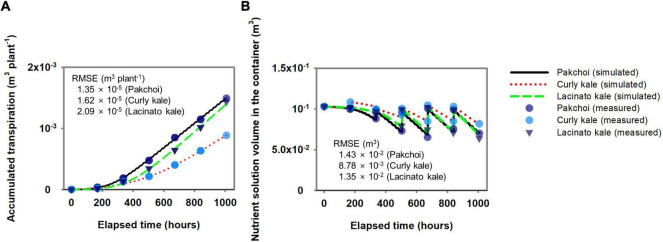
Comparison between simulated and measured accumulated transpiration **(A)** and subsequent changes of nutrient solution volume changes in the nutrient solution container **(B)** for verifying the simple hydroponic system model.

Using the measured data in Figure 2A, the transpiration parameters were estimated. The estimated transpiration parameters for pakchoi, lacinato kale, and curly kale simulated the amount of accumulated transpiration at the RMSE 1.35 × 10^–5^, 1.62 × 10^–5^, and 2.09 × 10^–5^ m^3^ plant^–1^, respectively (Figure 3A). The amount of accumulated transpiration was in the order, pakchoi > lacinato kale > curly kale, similar to the measurements. The calibrated transpiration model provided a good description of the water volume behavior in the nutrient container of pakchoi, curly kale, and lacinato kale at an RMSE of 1.43 × 10^–2^, 8.78 × 10^–3^, 1.35 × 10^–2^ m^3^ (Figure 3B). As explained in the “Materials and Methods” section, the water stored in the nutrient container was replaced weekly, and the simulation model showed the periodical replacement of reservoir water and subsequent water consumption by transpiration until the next water replacement.

**FIGURE 4:**
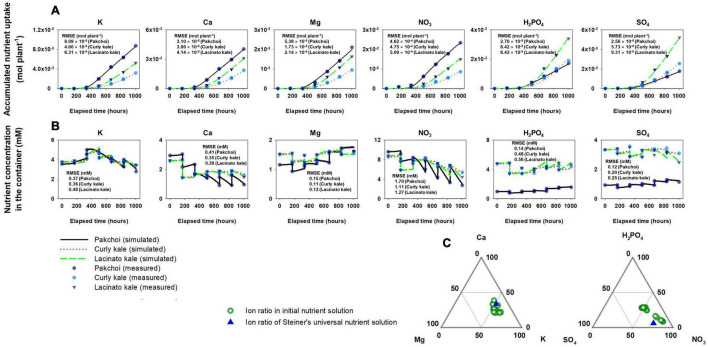
Comparison between simulated and measured accumulated nutrient absorption of three *Brassica* species **(A)** and subsequent nutrients molar concentrations in the hydroponic system’s nutrient solution container **(B)**. **(C)** Distribution of percentage molar ratio of cations and anions in the nutrient solution container’s initial nutrient solution after replacing the weekly used nutrient solution around the Steiner’s standard percentage molar ratios.

The estimated Michaelis-Menten parameters for nutrient absorption simulated the accumulated nutrient absorption with good agreement with the measured data (Figure 4A). RMSEs for the three species ranged from 4.60 × 10^–5^ to 8.09 × 10^–5^ mol plant^–1^ in K, 3.06 × 10^–5^ to 4.14 × 10^–5^ mol plant^–1^ in Ca, 1.73 × 10^–5^ to 5.38 × 10^–5^ mol plant^–1^ in Mg, 4.62 × 10^–4^ to 5.09 × 10^–4^ mol plant^–1^ in NO_3_, 2.70 × 10^–5^ to 8.42 × 10^–5^ mol plant^–1^ in H_2_PO_4_, and 2.56 × 10^–5^ to 9.31 × 10^–5^ mol plant^–1^ in SO_4_. With these nutrient absorption model calibrations, the simulated nutrient concentrations showed a good description of the measured nutrient concentration behavior in the water reservoir (Figure 4B). The percentage molar ratio of the initial nutrient conditions after the weekly used nutrient solution was distributed around Steiner’s standard percentage molar ratios (Figure 4C).

**FIGURE 5:**
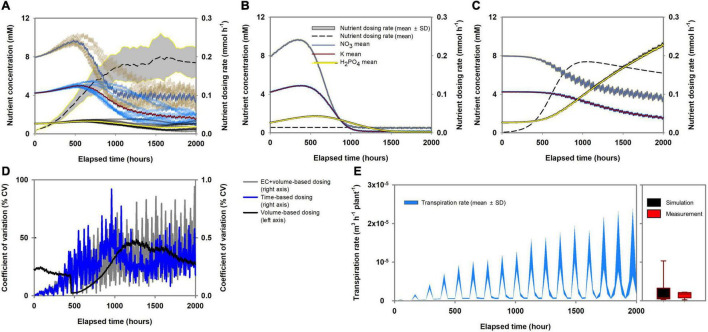
Characteristics of variations in root-zone nutrient concentration according to the nutrient dosing methods (pakchoi parameter applied simulation): **(A)** volume-based, **(B)** time-based, and **(C)** EC + volume-based methods. **(D)** The percent coefficient of variation of root-zone nutrient concentration according to the nutrient dosing methods. **(E)** Level of stochastic changes in the transpiration rate during the simulation analysis and box-plot comparing the distribution of the simulated transpiration rate (pakchoi parameter applied simulation) and the measured transpiration rate of pakchoi.

The effect of the three nutrient dosing methods (volume-, time-, and EC + volume-based) under stochastic transpiration variations is shown in Figures 5A–C. Here, the pakchoi model parameters, which have the highest absorption capacities for nutrients and transpiration, were used as a representative for analyzing the nutrient dosing effect. The root-zone response to volume-based dosing was not monotonic (Figure 5A). As the simulation proceeded, various routes of root-zone nutrient changes were simulated in volume-based dosing conditions. The EC + volume- and time-based methods showed reproducible and deterministic changes in the root-zone nutrients (Figures 5B,C). However, while time-based dosing showed similar variations to EC + volume-based dosing, overall decreasing tendencies in nutrient concentration were observed (Figure 5B). The percent coefficient of variation (% CV) summarizes the variation attributes of each dosing method (Figure 5D). The EC + volume- and time-based methods showed the lowest % CV. Figures 5A–C also illustrates the nutrient dosing rates. Volume-based dosing showed an irregular dosing rate, time-base dosing showed a constant dosing rate, and EC + volume-based dosing rate gradually increased as the simulation proceeded. During the simulation analysis, most of the stochastic transpiration rates generated by the random-walk cloud cover distributed within a similar range of the measured transpiration rate (Figure 5E).

**FIGURE 6:**
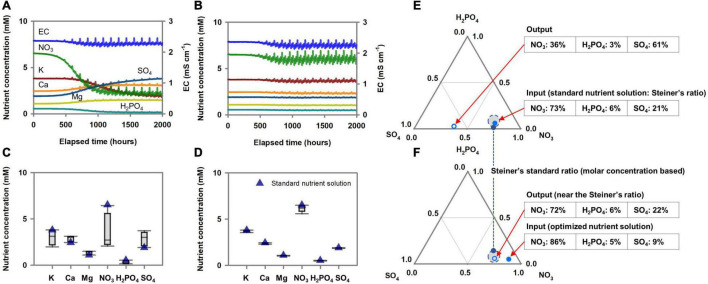
Optimization results of a standard nutrient solution for acquiring the reproducible and least deviating root-zone nutrient dynamics from the initial nutrient condition under the EC+volume-based nutrient dosing method. **(A)** Changes in root-zone nutrients concentration when the nutrient dosing was performed as a nutrient solution with a conventional standard nutrient solution. **(B)** Changes in root-zone nutrients concentration when the nutrient dosing was performed as an optimized nutrient solution. **(C)** Box-plot of the nutrient distribution during the nutrient dosing simulation with a nutrient solution with a conventional standard nutrient concentration. **(D)** Box-plot of the nutrient distribution during the nutrient dosing simulation with an optimized nutrient solution. **(E)** The percentage molar ratio’s final simulation output under the nutrient dosing condition of a conventional standard nutrient ratio input (Steiner’s ratio). **(F)** The percentage molar ratio’s final simulation output under the nutrient dosing condition of an optimized nutrient ratio input.

Figure 6 illustrates EC + volume-based dosing to control the root-zone nutrients according to the standard nutrient composition input. When the nutrient dosing composition was the same as the standard nutrient composition, the three *Brassica* species’ root-zone nutrient variations were reproducible and deterministic (Figure 6A), as shown in Figure 5C. However, they inevitably deviated from the initial nutrient composition (i.e., standard nutrient composition) (Figure 5C). On the other hand, the nutrient dosing composition acquired by optimization analysis to achieve minimal deviation of the root-zone nutrients from the standard composition provided approximately constant root-zone nutrients close to the initial nutrient conditions (i.e., standard nutrient composition) (Figures 6B,D). The conversion of the root-zone nutrient variations, optimized nutrient dosing composition, and the standard nutrient composition into mutual nutrient ratios displayed a plant stoichiometric scaling relationship among the nutrients (Figures 6E,F). The ternary graph of the percentage molar ratio of nutrients distinctively visualized effects of input nutrient ratio on the output nutrient ratio. Figure 6 also summarizes the relationship between the dosing nutrient composition and the standard nutrient composition. This result shows that a dosing nutrient composition identical to the standard nutrient composition does not result in standard nutrient conditions in the root-zone. The dosing nutrient compositions determined by the optimization analysis were nutrient solutions that least deviated from the standard nutrient conditions. The hydroponic systems in which the three *Brassica* species were cropped were simulated using the estimated nutrient uptake parameters.

**FIGURE 7:**
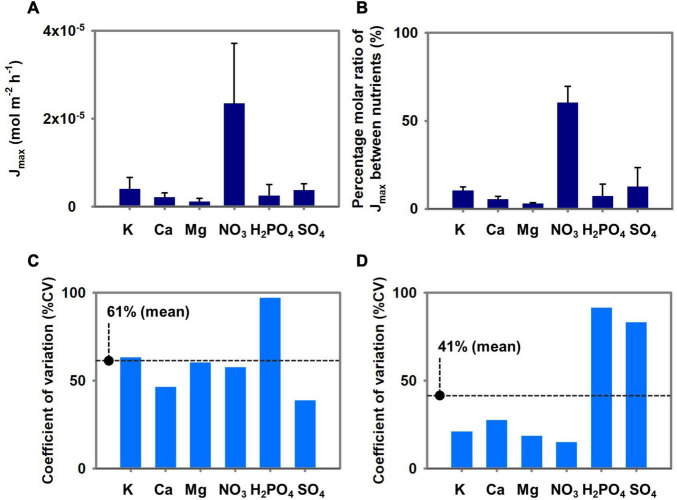
**(A)** Mean ± SD of *J*_*max*_ of three *Brassica* species (pakchoi, lacinato kale, and curly kale). **(B)** Percentage molar ratio between *J*_*max*_ of the nutrients. **(C)** The percent coefficient of variation among the *J*_*max*_ of three *Brassica* species. **(D)** The percent coefficient of variation among the percentage molar ratio between *J*_*max*_ of the nutrients.

Figures 7A,C illustrates the means, standard deviations, and % CV of the three *Brassica* species’ *J*_*max*_ parameters. Figures 7B,D shows the conversion of the *J*_*max*_ parameters to percentage molar ratio and summarizes the means, standard deviations, and % CV of the three *Brassica* species’ *J*_*max*_ ratios. The *J*_*max*_ for NO_3_ was the highest of three *Brassica*’s means, and the mean *J*_*max*_ for K, SO_4_, Ca, Mg, and H_2_PO_4_ followed in that order. However, relatively high % CVs were observed for K, Ca, Mg, NO_3_, and H_2_PO_4_ which differ the % CVs of percentage molar ratio of *J*_*max*_ between nutrients. The *J*_*max*_ conversion to percantage molar ratio reduced the % CV for K, Ca, Mg, NO_3_, and H_2_PO_4_, and increased that of SO_4_. Overall, the mean % CV for V_*max*_ was reduced from 61 to 41% when *J*_*max*_ was converted to percentage molar ratio.

## Discussion

Our theoretical analyses on nutrient dosing and root-zone nutrients provide insight into root-zone nutrient dynamics. Currently, nutrient dosing and time-dependent root-zone nutrient dynamics are considered as steady-state. However, by systematically varying the transpiration rate in the Michaelis-Menten nutrient absorption-based hydroponic system model, we could theoretically predict the appropriate nutrient dosing method for acquiring reproducible root-zone nutrient dynamics.

To our knowledge, only a few studies have theoretically analyzed root-zone nutrient dynamics in the hydroponic system. Heinen (1997) constructed a hydroponic simulation model and analyzed the system’s solute flux and distribution characteristics. Silberbush and Ben-Asher (2001) and Silberbush et al. (2005) developed a simplified hydroponic system model and analyzed nutrient concentration changes and ballast ion accumulation (Na and Cl). Hydroponic systems are essential for plant science and agricultural technologies because of their advantages in manipulating root-zone nutrients. However, root-zone nutrients are sensitive to perturbations. By elucidating plant stoichiometry and nutrient dynamics, root-zone nutrient dynamics offer new avenues for research and exploitation of dynamics in agricultural systems. However, little is known on the reproducibility of root-zone nutrient variations, which is essential for harnessing plant nutritional dynamics.

Our hydroponic system model with stochastic transpiration variation will enable us to examine the consequences of nutrient dosing in the root-zone. In addition to water absorption by plants, the root-zone nutrients could be affected at the microscopic scale. In the solute pathway from the external solution into root cells, ion diffusivity (Leitner et al., 2010), metabolic activity (Swift et al., 2020), and ion interaction (Munns and Tester, 2008) can affect nutrient influx, subsequently changing nutrient conditions in the root zone. However, in our study, we focused on the root-zone nutrient perturbation caused by transpiration since water absorption is a significant source of root-zone nutrients (Van Noordwijk, 1990; Le Bot et al., 1998). Macroscopic-scale nutrient variations caused by transpiration are frequently reported in in-field cultivation experiments (Shin and Son, 2016).

The reproducibility of nutrient dynamics determined by volume-, time-, and EC + volume-based dosing methods under stochastic transpiration variations revealed clearly distinct trends (Figures 5A–C). The reproducibility observed in the EC + volume- and time-based dosing methods suggests that the root-zone inconsistencies caused by the transpiration variations could be eliminated through periodic compensation of total nutrient absorption or a constant nutrient influx at the hydroponic system boundary. However, while the time-based periodical dosing showed reproducibility in terms of root-zone nutrients, the overall nutrients showed decreasing tendencies (Figure 5B). Notably, with time-based dosing, most nutrients displayed a steady-state. However, depending on the dosing interval or amount, the level of steady-state nutrients could be generally lower or higher than the initial root-zone nutrients. This observation is consistent with the findings of an agricultural experiment involving constant and periodical Mn and Zn dosing (Tzerakis et al., 2012). In the aforementioned study, Mn and Zn showed a gradual increase in the root-zone solution, leveling off after a certain period. In discrete time, a steady-state indicates that influx and efflux are equal in a system. A system that follows a Michaelis-Menten-based mechanism could have a steady-state solution (Golicnik, 2011). Thus, it could be concluded that the time-based nutrient dosing provided constant and periodical influx to the system, and the efflux, i.e., the nutrient absorption rate, approached the constant and periodical influx. However, in this case, the constant nutrient dosing could not follow the increasing nutrient absorption capacity of the plant. Therefore, fluctuations in the root-zone nutrients from their initial condition were observed.

As reported in previous studies, transpiration could destabilize the ratio between nutrient and water absorption; hence, root-zone nutrients significantly fluctuate along with water absorption variations (Van Noordwijk, 1990; Le Bot et al., 1998). The transpiration phenomenon is indicative of plant characteristics such as leaf area and stomatal conductivity; however, under normal growth conditions, transpiration is primarily driven by atmospheric environmental conditions (Pieruschka et al., 2010). Therefore, although we conducted experiment under controlled environmental conditions, the stochastic aspects between atmospheric dynamics and transpiration need to be considered in the simulation analysis. The root-zone nutrient variations observed under volume-based dosing conditions suggest the method is unsuitable for providing reproducible root-zone nutrient conditions. In the present study, we created stochastic transpiration variation by random-walk cloud cover in a periodic solar radiation model, revealing that although transpiration is a periodic phenomenon, root-zone nutrients will diverge even under stochastic fluctuations within the transpiration cycle. The volume-based dosing rate is determined by the water volume removed from the root-zone (Signore et al., 2016) while the standard nutrient composition is transported by the water flux to the system. Consequently, nutrient influx varies with transpiration variation and an irregular nutrient influx applied at the system boundary of the root-zone. Such root-zone nutrient behaviors has been reported in a study involving volume-based nutrient dosing (Signore et al., 2016). The root-zone nutrients displayed a wide range of variation when the nutrient dosing treatments were performed based on the water volume, indicating that root-zone nutrients can follow different time-series changes depending on the transpiration variations.

Similar to time-based dosing, EC + volume-based nutrient dosing generated reproducible time-series changes in the root-zone nutrients (Figure 5C). The dosing rate gradually increased as the simulation proceeded, indicating that the EC + volume-based nutrient dosing compensated for the increasing capacity for total nutrient absorption by plants. In contrast to the time-based nutrient dosing, nutrient dosing rates varied with plant growth; the total nutrients in the root-zone removed by the plant were periodically compensated by dosing. The kinetics of nutrient transport in plant roots are mostly determined by nutrient transporters (Swift et al., 2020). Therefore, total nutrient absorption approximately follows the influx kinetics of the total nutrients into the root cells. On the kinetics of nutrient transport in plant roots, nutrient selectivity could be conceptualized as the individual nutrient influx as regulated by the proportion of each nutrient’s transporter on the root surface and transporter affinity (Menge et al., 2011). Therefore, periodical compensation of total nutrient absorption by EC + volume-based dosing could reveal the proportion of nutrient absorption transporters.

Consequently, both constant periodical time-based and EC + volume-based dosing minimize the perturbation caused by transpiration variations and reveal the kinetics aspect of nutrient absorption. However, time-based dosing does not account for the plant’s increasing nutrient absorption capacity in dosing equations. As a result, reproducibility but with overall decreases or increases from the initial root-zone variation is observed depending on the dosing interval or amount (Figure 5B). In contrast, EC + volume-based dosing periodically matched the total nutrient influx to the root cell. From a theoretical perspective, these results suggest that this dosing practice can eliminate the two unknown variables effects (transpiration and total nutrient absorption variations) from the root-zone system boundary. Therefore, this indicates that the uncertainty in the root-zone nutrient dynamics could be filtered down to the level of the relative variations between the proportions of Michaelis-Menten parameters. Accordingly, the dosing nutrient composition for minimal deviation of root zone nutrients from the standard nutrient composition could be acquired by an optimization analysis (Figures 6B,D,F). Specifically, investigating optimal nutrient proportions in the dosing nutrient solution, which result in root-zone nutrients deviating the least from the standard nutrient composition, corresponds to a process for matching dosing nutrient proportions to the Michaelis-Menten parameter proportions.

This new perspective suggests that an optimization analysis can be used as a novel method to determine the nutrient dosing composition for generating the standard nutrient conditions in the root-zone. Conventionally in plant science, the standard nutrient solution has been routinely supplied to establish standardized root-zone nutrient conditions (De Rijck and Schrevens, 1998a). However, our simulation analysis indicates that a difference in nutrient dosing practice can lead to alterations in root-zone nutrients even when dosing with standard nutrient compositions. Furthermore, in the EC + volume-based dosing, a dosing nutrient composition identical to the standard nutrient composition does not result in standard nutrient conditions in the root-zone (Figures 6A,C,E). However, based on its reproducibility and proportional approach to the dosing nutrient composition and the Michaelis-Menten parameters, a dosing nutrient composition that results in minimal deviation of the root zone nutrients from the standard nutrient composition could be acquired (Figures 6B,D,F). Thus, in an on-site hydroponic system, a standardized and simplified nutrient management process might also be deduced from this perspective; for example, in a hydroponic system in operation, simple feedback of the nutrient proportion differences between the used solution and target composition proportion into the next dosing solution could expect the formation of stabilized root zone nutrient variability centered on the target proportion.

The variation in the Michaelis-Menten nutrient uptake parameters and how they affect the root-zone nutrients were not addressed as this was beyond the scope of the present study. However, we note the scaling relationships of nutrient and parameter proportions considered in our study. In the experiment, pakchoi, lacinato kale, and curly kale’s nutrient absorption capacity varied widely among the species (Figures 2B, 4A). On the contrary, significant reductions in % CVs were observed when the *J*_*max*_ of the three *Brassica* species were converted into a ratio between K, Ca, or Mg, NO_3_, and H_2_PO_4_ ‘s *J*_*max*_ (Figure 7D). On the other hand, the % CV of the *J*_*max*_ proportion increased with SO_4_. These proportion variations resulted in the stoichiometric proximity of K, Ca, Mg, NO_3_, and H_2_PO_4_, in the three *Brassica* species. We studied three *Brassica* species, and these are not meant to represent all vegetable species. However, the relative proportion of plant nutrients has been examined by plant stoichiometry, providing a scaling relationship of nutrient balances from plant nutrient variations (Parent et al., 2013). In the past, many standard nutrient solutions have been developed and widely used for plant science and agricultural cropping. These nutrient solutions have some differences in macronutrient concentrations; however, for most of them, the relative proportions of cations and anions indicate stoichiometric proximity (De Rijck and Schrevens, 1998a). Nevertheless, the mechanisms of stoichiometric homeostasis and the nutrient solutions’ physiological costs remain under investigation (Rouached and Rhee, 2017). The scaling relationship observed from a stoichiometric perspective across plant systems by the proportion approaches suggests a potential for an integrated framework for plant nutrient dynamics research.

## Conclusion

Our simulation analysis provided some clarity regarding root-zone nutrient dynamics using Michaelis-Menten parameters. Subsequently, based on the reproducibility of nutrient dosing, our simulation analysis predicted a proportion approach to the dosing nutrient composition and Michaelis-Menten parameters as a novel method of manipulating root-zone nutrient dynamics. This may turn conventional nutrient management practices into a simplified optimization problem. From the reduced complexity, we can expect to have a theoretical background to build a seamless framework for exploiting plant nutrient dynamics in the agronomic field and plant research. The framework presented here may provide a platform extended to fertilizer-emission-free agricultural production and plant stoichiometric research.

## Data Availability Statement

The raw data supporting the conclusions of this article will be made available by the authors, without undue reservation.

## Author Contributions

TA and JL designed the research. J-EP and GY performed the research. JJ, SK, and HK analyzed the data. TA wrote the article. All authors contributed to the article and approved the submitted version.

## Conflict of Interest

The authors declare that the research was conducted in the absence of any commercial or financial relationships that could be construed as a potential conflict of interest.

## Publisher’s Note

All claims expressed in this article are solely those of the authors and do not necessarily represent those of their affiliated organizations, or those of the publisher, the editors and the reviewers. Any product that may be evaluated in this article, or claim that may be made by its manufacturer, is not guaranteed or endorsed by the publisher.
